# Adapting to Smoke-Free Psychiatric Care: An Audit of Patient Behaviour, Medication Management, and Staff Challenges

**DOI:** 10.1192/bjo.2025.10625

**Published:** 2025-06-20

**Authors:** Praveen Kumar, Caio Bezzerraculas, Ananya Santosh, Nikki Thomson

**Affiliations:** 1 City Hospital, Aberdeen, United Kingdom; 2 New Craig’s Psychiatric Hospital, Inverness, United Kingdom

## Abstract

**Aims:** The audit conducted at New Craig’s Psychiatric Hospital’s Intensive Psychiatric Care Unit (IPCU) aimed to evaluate the impact of the Smoke-Free Perimeter Law on patient care and staff well-being. It sought to understand how the law affected patient behaviour and health, particularly for those on medications like clozapine, and to assess the changes in staff workload and safety. The study also aimed to identify the operational challenges and necessary adaptations in the IPCU following the law’s implementation.

**Methods:** Utilizing a mixed-methods approach over three months, the audit incorporated both quantitative analysis of patient records and qualitative data from staff surveys. Quantitatively, the focus was on incidents requiring medical intervention, medication administration, and staffing challenges. Qualitatively, a comprehensive survey was distributed to IPCU staff, featuring structured and open-ended questions to capture insights into the impact of the law, challenges faced, and suggestions for improvement.

**Results:** Patient-Related Outcomes: The analysis of 58 incidents revealed a prevalence of agitation, with lorazepam being the most administered medication. A notable case involved a patient whose clozapine levels were affected due to changes in smoking habits, leading to increased psychiatric symptoms. This highlights the complex interplay between lifestyle factors, like smoking, and medication efficacy.

The data indicated diverse triggers for patient incidents, suggesting varied patient reactions to the smoking restrictions.

Staff survey: All nine respondents reported increased workload, primarily attributed to managing the new smoking restrictions. Staff observed changes in patient behaviour and experienced heightened stress, linking these to the law’s enforcement.

Significant challenges were reported in accompanying patients for smoking breaks, suggesting an added operational burden.

**Conclusion:** The audit findings illuminate the multifaceted impact of the Smoke-Free Perimeter Law on both patients and staff in the IPCU. The increased frequency of incidents, particularly agitation, and the case of altered clozapine levels due to changes in smoking habits, underscore the intricate relationship between psychiatric medication management and patient lifestyle choices. Staff reported an escalation in workload and stress, suggesting that the law’s implementation has significantly altered the operational landscape of the IPCU. These outcomes suggest that while the intent of the law aligns with public health objectives, its application in a psychiatric care setting requires careful consideration of patient and staff needs. Future policy implementations should incorporate flexible, patient-centred approaches to ensure the well-being of all parties involved.

